# MEASURING MINDFULNESS IN OLDER ADULTS: A LITERATURE REVIEW OF EXISTING MEASURES

**DOI:** 10.1093/geroni/igz038.1136

**Published:** 2019-11-08

**Authors:** M Lindsey Jacobs, Jacqueline Gurevitch, Casey Catlin, Patricia M Bamonti

**Affiliations:** 1 VA Boston Healthcare System, Brockton, Massachusetts, United States; 2 VA Boston Healthcare System, Boston, Massachusetts, United States

## Abstract

Mindfulness is the process of non-judgmental, present-moment awareness and has increasingly been used in psychotherapy. Self-report measures that assess mindfulness are vital in clinical research and practice in order to determine the effectiveness of mindfulness-based psychotherapies. Many mindfulness measures have been developed, but it is unclear which measures have been validated with older adults. The purpose of this review was to identify measures that have been validated in adults age 50 and older. PubMed was searched through March 2019. Search terms were “mindfulness” and terms denoting measurement (e.g., “measure,” “measurement”). Review articles, dissertations, and non-English publications were excluded. Articles were independently evaluated by three raters. Studies describing measures that did not exclusively evaluate mindfulness were excluded. Sixty-two articles were included and described 27 mindfulness measures. The most frequently studied measures were the Five Facet Mindfulness Questionnaire (n=13) and the Mindfulness Attention Awareness Scale (n=14). Only three psychometric studies had a participant sample with a mean age of 50 and over, with one study having a sample with a mean age of 71. The measures evaluated in these studies were the Five Facet Mindfulness Scale and Langer Mindfulness Scale. Eighteen studies included some older adults, though the numbers were not large enough to yield a mean age over 50. Validation of mindfulness measures in adults age 50 and over has been largely neglected. Additional psychometric research is needed to validate commonly used measures in this population.

